# Interplay between plasmon and single-particle excitations in a metal nanocluster

**DOI:** 10.1038/ncomms10107

**Published:** 2015-12-17

**Authors:** Jie Ma, Zhi Wang, Lin-Wang Wang

**Affiliations:** 1 Joint Center for Artificial Photosynthesis and Materials Sciences Division, Lawrence Berkeley National Laboratory, Berkeley, California 94720, USA; 2 Materials Sciences Division, Lawrence Berkeley National Laboratory, Berkeley, California 94720, USA

## Abstract

Plasmon-generated hot carriers are used in photovoltaic or photochemical applications. However, the interplays between the plasmon and single-particle excitations in nanosystems have not been theoretically addressed using *ab initio* methods. Here we show such interplays in a Ag_55_ nanocluster using real-time time-dependent density functional theory simulations. We find that the disappearance of the zero-frequency peak in the Fourier transform of the band-to-band transition coefficient is a hallmark of the plasmon. We show the importance of the *d*-states for hot-carrier generations. If the single-particle *d*-to-*s* excitations are resonant to the plasmon frequency, the majority of the plasmon energy will be converted into hot carriers, and the overall hot-carrier generation is enhanced by the plasmon; if such resonance does not exist, we observe an intriguing Rabi oscillation between the plasmon and hot carriers. Phonons play a minor role in plasmonic dynamics in such small systems. This study provides guidance on improving plasmonic applications.

## Abstract (web summary)

Plasmons can enhance hot-carrier generation for efficient photochemical reactions, but the interplay between plasmons and single-particle excitations are difficult to capture in models. Here, the authors use real-time time-dependent density functional theory to study these interactions in silver nanocrystals.

## Graphical abstract

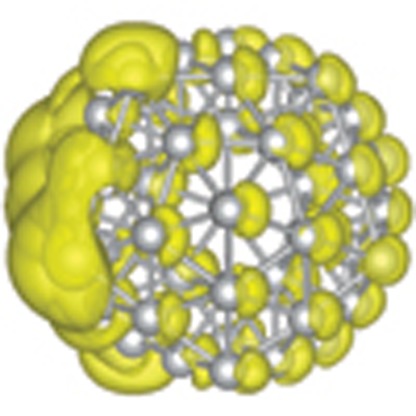

Plasmons are collective oscillations of the electron density, which can convert light energies into electronic excitations^1^,^2^. Due to the enhanced electromagnetic fields near metallic surfaces, metal nanostructures have been used in single-molecule spectroscopy, surface-enhanced Raman spectroscopy and surface-enhanced fluorescence^3^,^4^,^5^. For noble-metal nanostructures, the plasmon frequencies, which depend on the size, shape, metal composition and surrounding dielectric environment^6^,^7^, are generally in visible or ultraviolet regions that cover a large portion of the solar spectrum^8^. Due to the large oscillator strengths of plasmons, metal nanostructures can be used as antennas to absorb sunlight^1^,^8^,^9^. An intensely studied current topic is to use plasmons for photovoltaic or photochemical reactions. To realize those goals, the collective plasmon must be converted into single-particle excitations, that is, hot carriers, so that the carriers can be collected in solar cells or injected into chemical reactions^9^,^10^,^11^,^12^,^13^,^14^,^15^,^16^. However, some basic questions are yet to be answered. For example, how much light energy is absorbed by plasmons and hot carriers? Will all the plasmon energy convert to hot carriers and enhance the hot-carrier generation rates?

Plasmon modes can be simulated classically using the Drude model and Maxwell equations. Such calculations can yield accurate plasmon modes for systems down to tens of nanometres. However, when the system sizes shrink to the order of nanometres, the classical method could fail^17^,^18^ due to the lack of nonlocal response in the dielectric function and interband transitions. In such small systems, quantum mechanical descriptions, such as the linear-response theory, become necessary^17^,^19^,^20^,^21^,^22^. Quantum mechanically, the plasmon modes can be defined by the poles in the complex frequency-space *ω* of the inverse dielectric function *ɛ*^−1^(**r**_1_, **r**_2_, *ω*)^23^; specifically, a plasmon mode *ν*(**r**, *ω*_p_) satisfies ∫*ɛ*(**r**_1_, **r**_2_, *ω*_p_)*ν*(**r**_2_, *ω*_p_)d**r**_2_=0. Here the plasmon frequency *ω*_p_ is a complex number, with its imaginary part indicating the plasmon life time. Unfortunately *ɛ*^−1^(**r**_1_, **r**_2_, *ω*) does not provide a real-time picture of the interplays between the plasmon and hot carriers. There are several recent theoretical studies on the plasmon-induced hot-carriers generations, showing that hot carriers can be effectively excited by plasmons^24^,^25^. However, those studies treated plasmons classically as oscillating electromagnetic fields, and used the perturbation theory (Fermi's golden rule) to calculate the hot-carrier generations. They did not describe the feedback of hot carriers to plasmons, nor represent the plasmon and single-particle excitations in a unified quantum mechanical framework.

The real-time (rt) time-dependent density functional theory (TDDFT) is a powerful tool to study the plasmonic processes. The rt-TDDFT describes directly the Coulomb interactions between electrons, and its ability to describe plasmons has been demonstrated in many previous publications^26^,^27^,^28^,^29^,^30^. The rt-TDDFT was used to study jellium models or small systems such as one-dimensional atom chains^26^,^27^,^30^. There were also rt-TDDFT simulations on nanoclusters up to several hundred atoms using local-orbital basis or real-space grids^28^,^29^. However, those works studied only the optical absorption spectra^28^,^29^, instead of the interplays and energy transfers between the plasmon and single-particle excitations. For many interesting physical problems, including the plasmon decay, hot-carrier generations and carrier transports, long-time simulations (10–1,000 fs) are needed. Our recently developed fast rt-TDDFT algorithm^31^ enables us to simulate such long time with plane-wave basis sets.

In this work, we will use rt-TDDFT to study the plasmon dynamics in a Ag_55_ nanocluster. The Ag_55_ nanocluster has been experimentally synthesized and its atomic structure is well determined^32^,^33^. A recent experiment measured the plasmon frequency of 3.8 eV (ref. 34). Such small nanoclusters may have great potentials for photocatalysis due to high carrier-injection rates. We propose an unambiguous approach to distinguish the plasmon from single-particle excitations based on the band-to-band transition coefficient. We find that if the single-particle *d*-to-*s* excitations resonant to the plasmon frequency exist, most plasmon energy will be converted into hot carriers, enhancing hot-carrier generations. However, if the resonances do not exist, the plasmon decays slowly and may exhibit Rabi oscillations. We propose that by modifying the electronic structures of nanoclusters (for example, the number of electrons, plasmon frequency, *d*-band energy and so on), the plasmon decay and hot-carrier generations can be tuned. The electron–phonon interactions play a minor role during the plasmon decay in Ag_55_.

## Results

### Laser excitations in Ag_55_

**Figure 1:**
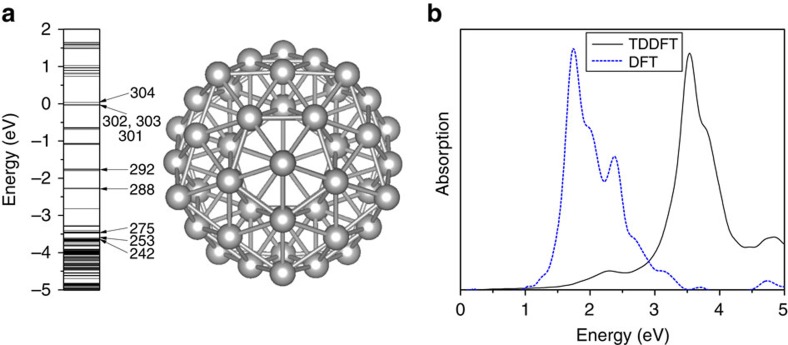
The calculated eigen energies, atomic structure and absorption spectra of Ag_55_. (**a**) The calculated atomic structure and eigen energies of the icosahedral Ag_55_. The Fermi energy is set to zero. Several important states are labelled. Each silver ball represents a Ag atom. (**b**) The absorption spectra calculated by the rt-TDDFT (the black-solid line) and by the DFT eigen states (the blue-dashed line). The heights of the main peaks are normalized.

The calculated atomic structure and eigen energies of Ag_55_ are shown in Fig. 1a. The neutral Ag_55_ has 605 electrons and is an open-shell system. The 301st–303rd states are almost degenerated and partially occupied, and the 304th state is the lowest unoccupied state (LUMO). To study the excitations, we apply an external electric field 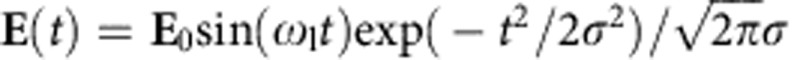 to mimic a laser pulse at the frequency *ω*_l_ with a Gaussian envelope. The optical absorption spectrum can be obtained from the time-dependent dipole response (the imaginary part of *α*(*ω*) described in Methods section), and is shown in Fig. 1b. We observe a strong absorption peak at *ω*_p_≈3.6 eV, which is in good agreement with the recent experimental result of 3.8 eV (ref. 34). We have also calculated the absorption spectrum from the DFT single-particle eigen states: 
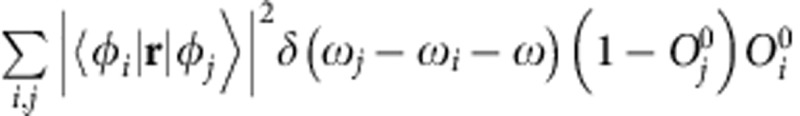
, where *φ*_*i*_, *ω*_*i*_ and 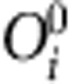 are the wave function, eigen energy and Fermi-Dirac occupation of the *i*th eigen state, respectively. The resulting curve shows a main peak at ∼1.7 eV (Fig. 1b). In all the calculations, we have modified the pseudopotential, so that the calculated Ag *d*-band energy agrees with the experiment^35^, which is shown in Supplementary Note 1 and Fig. 1.

**Figure 2:**
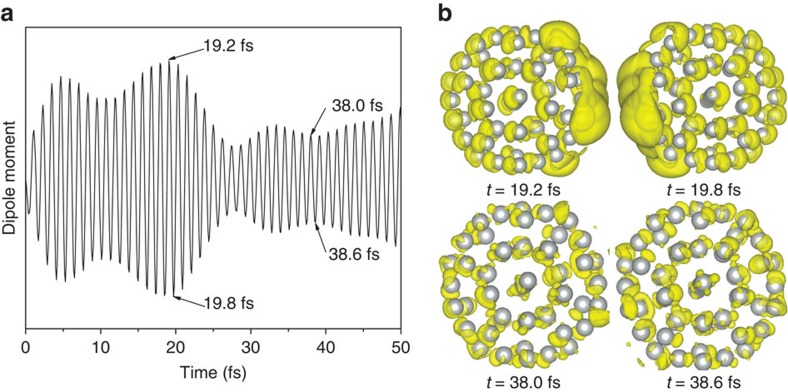
The dipole moment and charge density oscillations. (**a**) The dipole moment oscillations as a function of time. (**b**) The charge density difference Δ*ρ* when the dipole moment achieves a local maximum or local minimum as labelled in **a**. At *t*=19.2 and 19.8 fs, the dipole moment oscillation amplitude is large. The Δ*ρ* is localized on the surface, and sloshes from one side of the nanocluster to the other side, fitting well with the classical picture of the surface plasmon mode. When the dipole moment oscillation is decayed (*t*=38.0 and 38.6 fs), the charge sloshing disappears, and Δ*ρ* is no longer localized on the surface.

To exam the resonant excitation, we set the laser frequency *ω*_l_=*ω*_p_ with *σ*=6 fs and |**E**_0_|=10^−3^ Hartree per Bohr. The electric field is along the *x* axis, starting at *t*=0 fs and fading off after ∼10 fs. In response to this external electric field, the dipole moment of the Ag_55_ nanocluster oscillates along the *x* axis, as shown in Fig. 2a. From 0 to 10 fs, as the electric field exists (the laser is on), the charge density is driven by the external electric field, and both the plasmon and single-particle modes have been excited. After 10 fs, as the external electric field fades off, the dipole oscillation continues by itself due to internal excitations. From 10 to 20 fs, the dipole oscillation amplitude is rather large. Two of the charge sloshing pictures Δ*ρ*(**r**, *t*)=*ρ*(**r**, *t*)−*ρ*_0_(**r**) (where *ρ*_0_(**r**) is the ground-state charge density) at *t*=19.2 and 19.8 fs are shown in Fig. 2b. The Δ*ρ* is localized on the surface, and sloshes from one side of the nanocluster to the other, fitting well with the classical picture of the surface plasmon. From 20 to 27 fs, the dipole oscillation amplitude decays to relatively small values. Two of the charge density differences Δ*ρ* at *t*=38.0 and 38.6 fs are shown in Fig. 2b. The Δ*ρ* is not localized on the surface and does not slosh, which may suggest the plasmon decay qualitatively. Such dipole oscillations can be observed experimentally as demonstrated in a recent experiment by Mittal *et al*.^36^ Using optical pump and probe method, they observed the polarization oscillations up to 80 fs in Ag nanoclusters. Their experiments confirmed the possibility of long-living dipole oscillations, similar to our results.

### Distinguishing the plasmon mode

How to distinguish the collective plasmon excitation from the non-collective single-particle excitation quantum mechanically is a challenging issue. In particular, for small molecules and nanostructures, the plasmon frequency *ω*_p_ overlaps with the single-particle excitation energies, which makes their interplays interesting but difficult to analyse. There are several previous publications on this topic using different approaches^11^,^23^,^37^,^38^,^39^,^40^,^41^,^42^,^43^. The plasmon and single-particle excitations cannot be clearly distinguished from the absorption spectrum, because they both can have strong absorption peaks for small nanoclusters. Beck identified the plasmon using charge density oscillations^43^. However, the resonant but non-collective single-particle excitation from the *i*th eigen state to the *j*th eigen state also produces a charge density oscillation. This can be seen from the expansion of the time-dependent wave function *ψ*_*i*_(*t*) on the eigen states in a perturbation theory:


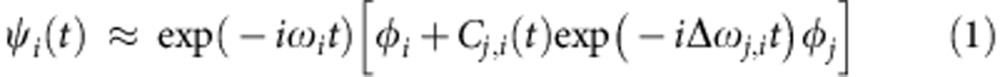


and the charge density change is


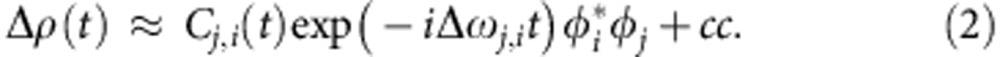


For resonant excitations, Δ*ω*_*j*,*i*_=*ω*_*j*_−*ω*_*i*_=*ω*_p_, so Δ*ρ*(*t*) also oscillates at the frequency *ω*_p_. Thus, we cannot use the charge density (or dipole moment) oscillation alone to tell the difference. One can attempt to use the charge density plot, such as Fig. 2b, to say there may be stronger plasmon excitations at 19 fs than those at 38 fs, but it is difficult to quantify the amounts of the plasmon and single-particle excitations. Krauter *et al*.^41^ proposed to identify plasmons based on orbital transitions and momentum conservations, but their approach requires certain (quasi-)symmetry of the system. Other groups proposed to identify plasmons by scaling the electron–electron interaction^23^. Although useful, this approach requires a serial of calculations on artificial systems. The occupation numbers on eigen states (that is, 
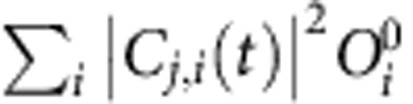
) were also used to identify plasmons^30^,^40^. However, the occupation change may be a mixture of both the plasmon and single-particle excitations, as we will show below. We find the transition coefficient *C*_*j*,*i*_(*t*), especially the Fourier transform of *C*_*j*,*i*_(*t*) (instead of 
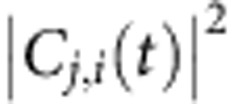
, which loses the phase information), can be used to distinguish the plasmon from single-particle excitations in an unambiguous way.

**Figure 3:**
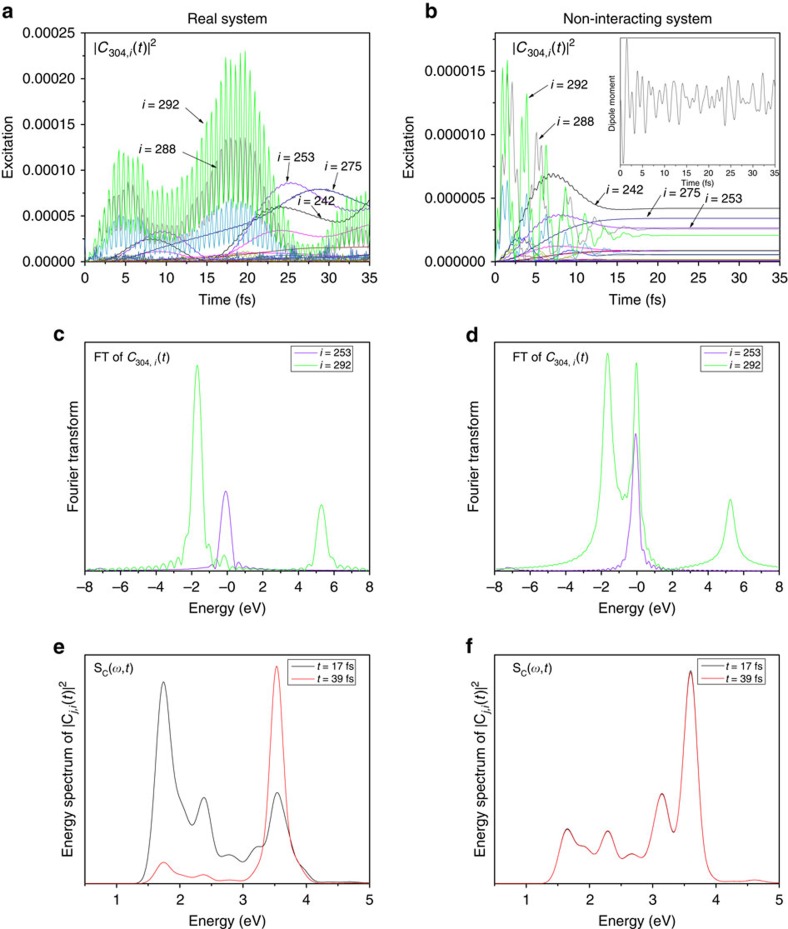
The analysis of the transition coefficients *C*_*j*,*i*_(*t*). We show *j*=304 (LUMO) state here. (**a**) The 
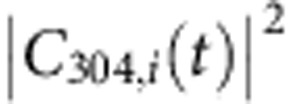
 indicates the transition from the *i*th state to the LUMO (304th) state. Several important transitions are labelled. Two distinct types (rapidly oscillating and slowly varying) of transitions can be observed. (**b**) The 
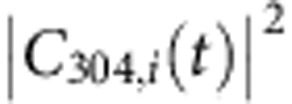
 for the non-interacting system. The slowly varying transitions behave similarly to those in **a** but the rapidly oscillating ones are different. The inset shows that the dipole moment oscillates irregularly. (**c**) The Fourier transform (FT) of *C*_*j*,*i*_(*t*) for the real system. There is no peak at 0 for the rapidly oscillating transition (*i*=292). (**d**) The FT of *C*_*j*,*i*_(*t*) for the non-interacting system. There is a peak at 0 for the oscillating transition (*i*=292). (**e**) The energy spectrum of 
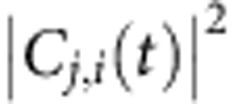
 (*S*_C_(*ω*, *t*)) at *t*=17 and 39 fs for the real system. At *t*=17 fs, there are strong off-resonant peaks at 1.7 and 2.3 eV; at *t*=39 fs those peaks disappear and the resonant peak at 3.6 eV is enhanced. (**f**) The *S*_C_(*ω*, *t*) for the non-interacting system. The main peak is always at 3.6 eV with no significant off-resonant peaks.

Equations (1) and (2) are general formulas that apply for all transitions. However, the collective behavior of the plasmon must have caused unique feature of *C*_*j*,*i*_(*t*), compared with *C*_*j*,*i*_(*t*) of single-particle excitations. In the following we will take *j*=304 (LUMO) state as an example to discuss the behaviours of *C*_*j*,*i*_(*t*). Figure 3a shows 
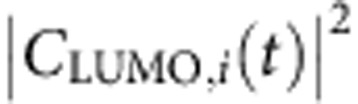
 for all the transitions from the occupied states (*i*≤303) to the LUMO state (the 304th state). We observe two clearly distinct types of transitions. One type of 
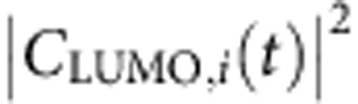
 shows regular high-frequency synchronized oscillations (for example, *i*=292), and the other shows smooth amplitude variations (for example, *i*=253).

All the slowly varying transitions have nearly resonant energies *ω*_LUMO_−*ω*_*i*_≈*ω*_p_ (for example, *ω*_LUMO_−*ω*_275_=3.5, *ω*_LUMO_−*ω*_253_=3.65 and *ω*_LUMO_−*ω*_242_=3.7 eV). The Fourier transform of *C*_LUMO,253_(*t*) (Fig. 3c) shows only one peak around *ω*=0. Thus, we have *C*_*j*,*i*_(*t*)=*f*(*t*), where *f*(*t*) is a slowly varying (compared with *ω*_p_) function, and equation (1) can be rewritten as:


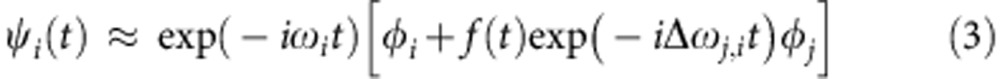


This is a typical independent single-particle excitation described by the single-particle time-dependent perturbation theory within the rotating-frame approximation. Only single-particle transitions with nearly resonant frequencies Δ*ω*_*j*,*i*_≈*ω*_p_ can be effectively excited. The charge density oscillates at Δ*ω*_*j*,*i*_≈*ω*_p_ due to the phase factor exp(−*i*Δ*ω*_*j*,*i*_*t*). The single-particle excitations correspond to the so-called ‘hot-carriers'.

In contrast, all the rapidly oscillating transitions have off-resonant energies *ω*_LUMO_−*ω*_*i*_<*ω*_p_ (for example, *ω*_LUMO_−*ω*_292_=1.78 and *ω*_LUMO_−*ω*_288_=2.3 eV), and the Fourier transform of *C*_LUMO,292_(*t*) shows two peaks at Δ*ω*_*j*,*i*_±*ω*_p_. Both the real and imaginary parts of *C*_LUMO,292_(*t*) (Supplementary Fig. 2 and Note 2) oscillate around zero, which confirms the disappearance of the zero-frequency peak. Because the Fourier transform has two peaks at Δ*ω*_*j*,*i*_±*ω*_p_, *C*_*j*,*i*_(*t*)=*A* exp[*i*(Δ*ω*_*j*,*i*_+*ω*_p_)*t*]+*B* exp[*i*(Δ*ω*_*j*,*i*_−*ω*_p_)*t*], and it can be written as 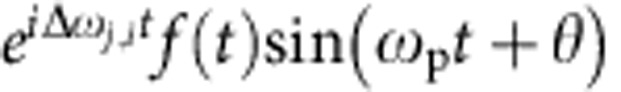. Here *A*, *B* and *f*(*t*) are slowly varying (compared with *ω*_p_) functions, and *θ* is a complex phase factor. Thus, equation (1) can be rewritten as:


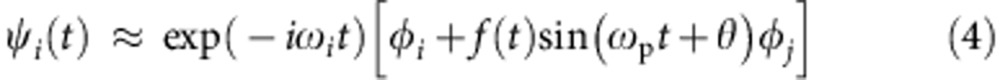


It cannot be described by the single-particle perturbation theory. It also does not require that the resonant condition Δ*ω*_*j*,*i*_≈*ω*_p_, as Δ*ω*_*j*,*i*_ does not appear in equation (4). The charge density oscillation (square of equation (4)) is contributed coherently from all the cross terms: 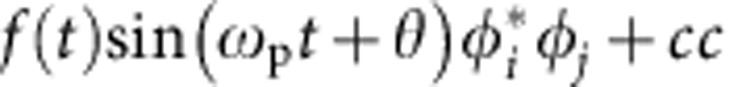, with the same frequency *ω*_p_. These synchronized off-resonant transitions constitute the plasmon mode. Since there is no resonant requirement for the plasmon-related transitions, many transitions with 
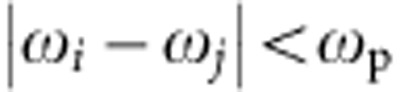
 can collectively and coherently construct the plasmon through electron–electron interactions. This contributes to the large plasmon oscillator strength, making it a strong light absorber. However, since the total oscillator strength is conserved, the non-collective single-particle *i* to *j* transitions are reduced. In Fig. 1b, compared with the strong single-particle excitations around 1.7 eV in the DFT spectrum, the TDDFT spectrum around 1.7 eV becomes much weaker. Most of their oscillator strengths have been shifted to the plasmon peak around 3.6 eV. The concept of plasmon as a collection of coherent transitions has long existed. Our analysis fits the picture well and provides direct evidence.

To further prove that the rapidly oscillating *C*_*j*,*i*_(*t*) comes from the plasmon, we have performed a time-dependent evolution on a non-interacting system with the same external electric field. In this simulation, we fix the potential in the Hamiltonian *V*[*ρ*(**r**, *t*), *t*]=*V*[*ρ*(**r**, 0), *t*] (except the external potential) making it independent of the charge density. We would like to stress that this non-interacting system is an unrealistic system with no experimental counterpart. In this model, all the electrons are treated as independent single particles. Because the electron–electron interaction is zero, the plasmon excitation is impossible. Thus, the plasmon-related transitions should behave differently, while the single-particle transitions should remain the same.

The dipole moment of the non-interacting system (inset of Fig. 3b) shows an irregular behavior, compared with Fig. 2a. For the slowly varying transitions (for example, *i*=253), both the 
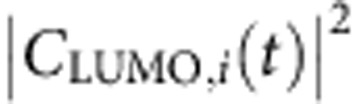
 (Fig. 3b) and the Fourier transform (Fig. 3d) are similar to those in Fig. 3a,c. However, for the rapidly oscillating transitions, the regular oscillations of 
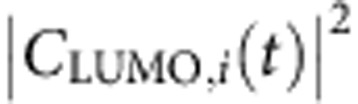
 (for example, *i*=292) are replaced by irregular oscillations. The irregularly oscillating transitions decay with time, and almost disappear after the laser fades off. Besides the two peaks at Δ*ω*_*i*,*j*_±*ω*_p_ in Fig. 3c, the Fourier transform of *C*_LUMO,292_(*t*) has a strong peak at *ω*=0 in Fig. 3d. This can be explained by the independent single-particle time-dependent perturbation theory^44^ under a sin(*ω*_p_*t*) perturbation along the *x* axis:


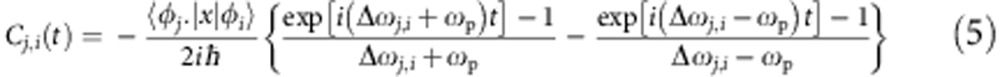


Apparently, the Fourier transform should have three peaks in agreement with those in Fig. 3d. If we substitute equation (5) into equations (1) and (2), besides the oscillations at the frequency *ω*_p_, there are extra oscillating terms at the frequencies Δ*ω*_*j*,*i*_. These off-resonant oscillations have different Δ*ω*_*j*,*i*_ and thus cannot add up in sync, which results in the irregular oscillations shown in Fig. 3b. To have synchronized plasmon oscillations, the *ω*=0 peak in the Fourier transform must disappear, which is a hallmark of the plasmon mode. The disappearance can only exist with interactions between different transitions.

In refs 23, 42, when there are no electron–electron interactions (*λ*=0 in ref. 23), the susceptibility *χ* equals to that of the non-interacting system *χ*^0^. Thus, their model system with no electron–electron interactions is exactly the same as our non-interacting system. The absorption spectrum of the non-interacting system is exactly the DFT result shown in Fig. 1b. (23) showed that the plasmon frequency significantly increases as *λ* increases. Similarly, we find that the main absorption peak of the real system (*λ*=1) is ∼1.9 eV higher than that of the non-interacting system (*λ*=0).

In the discussion above, we have used *i*=253 and 292 with *j*=LUMO to represent the two types of transitions, respectively. All other transitions can also be classified into the two distinct types. The 
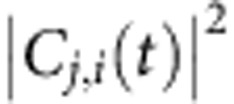
 for several other states are shown in the Supplementary Fig. 3 and discussed in Supplementary Note 3.

Here we summarize the analysis above. Both the plasmon and single-particle excitations are described by band to band transitions (equation (1)) in TDDFT. The single-particle excitation is the resonant transition (off-resonant transitions cannot be efficiently excited as single-particle excitations), and equation (1) is rewritten as equation (3); the plasmon is constituted of synchronized off-resonant transitions (the rotating-frame approximation cannot be used), and equation (1) is rewritten as equation (4). The disappearance of the zero-frequency peak in the Fourier transform of *C*_*j*,*i*_(*t*) due to the electron–electron interaction is a hallmark of the plasmon. For single-particle excitations, the charge density oscillation comes from the phase factor interference between *φ*_*i*_ and *φ*_*j*_ (Δ*ω*_*j*,*i*_≈*ω*_p_) and *C*_*j*,*i*_(*t*) does not oscillate; for plasmons the oscillation comes from the *C*_*j*,*i*_(*t*) amplitude oscillations. Each transition (*j*, *i*) can either belong to plasmon (off-resonant) or single-particle (near-resonant), but cannot belong to both. This provides an unambiguous way to sort out the plasmon from single-particle excitations.

### Energy transfers in Ag_55_

We have calculated the energy spectrum of 
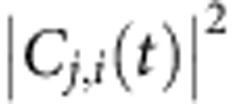
, defined as 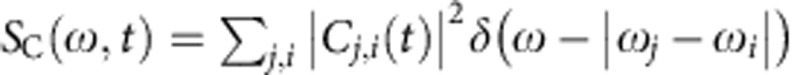 (*j* and *i* denote unoccupied and occupied states, respectively), which represents the number of transitions at the energy *ω* for a given time *t*. For the real system, as shown in Fig. 3e, *S*_C_(*ω*, *t*) has peaks around 1.5–2.5 eV at *t*=17 fs. Those off-resonant transitions constitute the plasmon. At *t*=39 fs, those off-resonant peaks disappear, and the resonant peak is strengthened, showing that the plasmon has been converted into resonant single-particle excitations (hot carriers). From *t*=17 to 39 fs, the laser is off. Thus, the hot carriers are generated completely by the plasmon. For the non-interacting system (Fig. 3f), *S*_C_(*ω*, *t*) shows much smaller peaks around 1.5–2.5 eV (no plasmons), while the main peak always appears at the resonant frequency (single-particle excitations). There is no change in *S*_C_(*ω*, *t*) from 17 to 39 fs because there are no laser nor electron–electron interactions.

**Figure 4:**
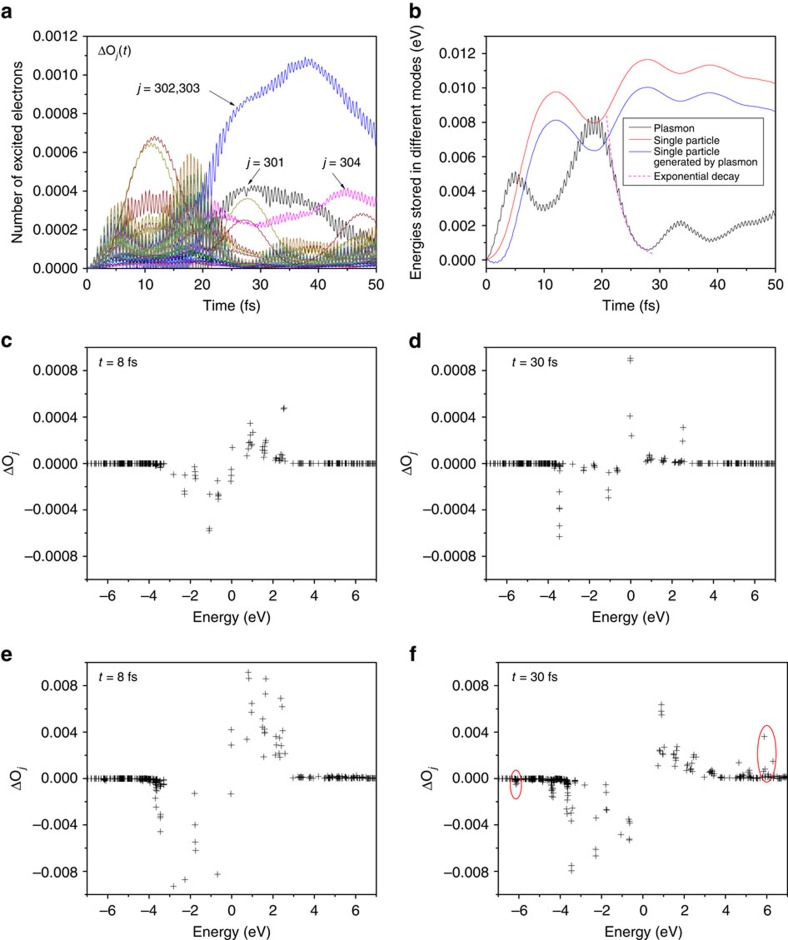
The time-dependent excitations in Ag_55_. (**a**) The number of excited electrons on every eigen state as a function of time. Several important eigen states are labelled. The major excitations are onto the 302nd and 303rd eigen states. (**b**) The energies stored in the plasmon and single-particle excitations. The blue line is the single-particle energy generated by the plasmon, which is defined as the energy difference in the single-particle mode between the real system (rt-TDDFT simulation) and the non-interacting system (in which plasmons cannot exist) under the same laser illumination. The pink-dashed line is an exponential decay of exp(−*ω*_p_*t*/*Q*) with *Q*=13.3. (**c**,**d**) The number of excited electrons (positive) and holes (negative) as a function of the corresponding eigen energy with respect to the Fermi energy at *t*=8 and 30 fs. At 8 fs, most excitations are around the Fermi energy and at 30 fs the excitations are concentrated in a few sharp peaks. (**e**, **f**) The number of excited carriers under a strong laser illumination. We observe some high-energy excitations at 30 fs (circled by red), which indicates nonlinear effects.

We have also calculated Δ*O*_*j*_(*t*) defined by equation (8) in Methods section, which is the occupation change on the *j*th eigen state. The numbers of excited electrons (positive Δ*O*_*j*_(*t*)) on all eigen states are shown in Fig. 4a. Because the occupation change on an eigen state *j* can have both resonant and off-resonant transitions from different states *i*, Δ*O*_*j*_(*t*) is a combination of both slowly varying and rapidly oscillating components as shown in Fig. 4a, different from *C*_*j*,*i*_(*t*) in Fig. 3. We find the major excited electrons are on the eigen states around the Fermi energy (for example, the 302nd and 303rd eigen states) after 20 fs. Thus, the single-particle excitations (resonant to the plasmon frequency) from low-energy *d*-states to the eigen states around the Fermi energy are the major plasmon decay (hot-carrier generation) channels.

Our analysis allows us to separate the energies stored in the plasmon and hot carriers (see Methods section), as shown in Fig. 4b. We also plot the single-particle energy generated by the plasmon, which is defined as the energy difference in the single-particle mode between the real system and the non-interacting system (no plasmons) under the same laser. After the laser is turned on at *t*=0, the light energy is absorbed by both the plasmon and resonant single-particle (hot-carrier) excitations, with almost equal rates. Compared with the non-interacting system, the single-particle energy of the real system is lower at first (the blue line is negative), indicating that less hot carriers are generated at first due to the plasmon. After 3 fs, the blue line becomes positive, indicating more hot carriers are generated due to the plasmon. After the laser fades off at *t*=10 fs, the energy in the plasmon mode does not decay immediately, and we even observe a small single-particle to plasmon energy transfer around *t*=17 fs. From 20 to 30 fs, the plasmon energy starts to decay and is almost completely transferred into single-particle excitations. The speed of the decay agrees with previous experiments that the plasmon decay timescale is 1–100 fs (refs 11, 36). By fitting the decay shoulder with an exponential function exp(−*ω*_p_*t*/*Q*), we obtain *Q*=13.3 (the pink-dashed line in Fig. 4b). Without the plasmon (the non-interacting system), the final energy stored in hot carriers is only ∼15% of that with the plasmon, showing that the plasmon can enhance the hot-carrier generations by ∼6.6 times in Ag_55_. In a recent experiment, Zheng *et al*.^45^ separated the plasmon-induced and photoexcited hot carriers in Au nanowires. With the transverse electric polarized laser, the plasmon was excited and the carriers excited by both plasmon and photons were collected through an Ohmic device; with the transverse magnetic polarized laser, the plasmon was not excited and only the carriers excited by photons were collected. In Fig. 3 of ref. 45, the ratio of excited carriers (transverse electric:transverse magnetic) is about 5:1 at the plasmon frequency. This measured plasmon-induced hot-carrier enhancement is in agreement with our result (about 6.6:1). Although the experiment was performed on Au systems, the agreement provides support for our results. We have performed the rt-TDDFT simulation up to 400 fs (Supplementary Fig. 4. and Note 4), and do not observe strong energy back-flows from hot carriers to plasmon.

The hot-carrier energy distributions at *t*=8 and 30 fs are shown in Fig. 4c,d. At *t*=8 fs, there are broad excitations around the Fermi energy, indicating the plasmon excitation. At *t*=30 fs, as the plasmon is decayed to hot carriers, the excited electrons and holes are concentrated in a few sharp peaks. These peaks can be classified into two groups. The major hot holes are around −3.6 eV with the corresponding electrons around the Fermi energy (*d* to *s* transitions). There are fewer hot holes at −1.1 eV with the corresponding hot electrons at 2.5 eV (*s* to *p* transitions). In a recent perturbation calculation of plasmon-induced hot-carriers in bulk Ag (ref. 24), the sharp peaks of the hot-carrier energy distribution were also observed. The major surface-plasmon-induced hot holes are at −3.6 eV with hot electrons around the Fermi energy^24^, which is in excellent agreement with our results. They also observed other hot holes at −0.1 and hot electrons at 3.6 eV, which qualitatively but not quantitatively agrees with our results. This is probably because the nanostructure strongly affects the nonlocalized *s* and *p* states but does not strongly affect the localized *d*-states.

Using rt-TDDFT, we can also simulate nonlinear effects, by increasing the laser field by 10 times. The calculated Δ*O*_*j*_ at *t*=8 and 30 fs are shown in Fig. 4e,f. At *t*=8 fs, the excitations are similar to those in Fig. 4c, indicating the plasmon excitation. However, at *t*=30 fs, we observe some high-energy excitations around ±6 eV (circled by red in Fig. 4f), which were not observed in Fig. 4d. Because the high-energy excitations do not exist before the laser fades off (*t*=8 fs in Fig. 4e), they are not due to the nonlinear laser excitation. The energies of those carriers (about 6 eV) are much greater than the plasmon frequency (3.6 eV), and thus the high-energy carriers are likely due to double excitations by the plasmon. The simulation predicts high-energy hot-carrier generations.

### Tuning the energy transfers

**Figure 5:**
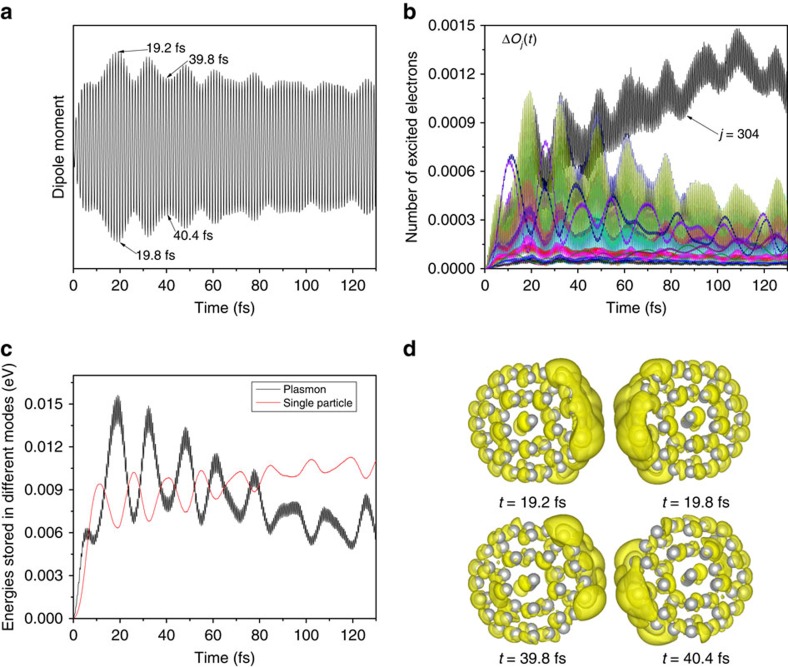
The time-dependent results for the negatively charged nanocluster. (**a**) The dipole moment shows a much slower decay compared with the neutral Ag_55_ (Fig. 2a). (**b**) The number of excited electrons Δ*O*_*j*_(*t*). The major electron excitations are onto the 304th eigen state. The excitations onto the 301st–303rd eigen states shown in Fig. 4a do not exist. (**c**) The energies stored in the plasmon mode and single-particle excitations. The energy transfer from the plasmon to single-particle excitations is also much slower compared with Fig. 4b, which indicates the slow plasmon decay, because the main single-particle *d* to *s* excitation channels resonant to the plasmon are blocked in 
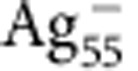
. (**d**) The charge density difference Δ*ρ* at time *t* as labelled in **a**, which corresponds to the local maximum or local minimum of the dipole moment. Compared with Fig. 2b, the significant charge sloshing on the surface still exists around 40 fs, which shows the plasmon is not fully decayed.

As mentioned above, the major hot-carrier generations in Ag_55_ are from the low-energy *d*-states to the eigen states around the Fermi energy (for example, 302nd and 303rd states). It is interesting to see what would occur if these transitions are blocked. We first consider the negatively charged 
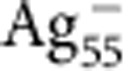
, which is a closed shell system. All the states *j*≤303 are fully occupied, and thus cannot accept electrons from *d*-states. The only important channel is the *d* to 304th state excitation. The amplitude of the dipole oscillation of 
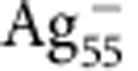
 (Fig. 5a) decays much slower than that of the neutral Ag_55_ (Fig. 2a). The numbers of excited electrons Δ*O*_*j*_(*t*) (Fig. 5b) confirm that the dominant hot electrons are on the 304th state. Because most hot-carrier generation channels are closed, the energy stored in the plasmon decreases slowly (Fig. 5c). After 100 fs the energy stored in the plasmon is still decaying and is not totally transferred to hot carriers. We observe small energy transfers and oscillations between the plasmon and single-particle excitations, and the same oscillations of the dipole moment (Fig. 5a). The charge density differences Δ*ρ* (Fig. 5d) at *t*=19.2 and 19.8 fs are similar to those of the neutral Ag_55_ (Fig. 2b). At *t*=39.8 and 40.4 fs, there are still strong charge sloshings on the surface, different from those of the neutral Ag_55_, which also suggests the slow plasmon decay. We would like to stress that the striking change due to one additional electron is related to the electric structure of Ag_55_. By adding one electron, most hot-carrier generation (plasmon decay) channels are blocked in 
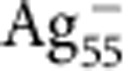
, and thus the plasmon decay becomes much slower. However, for large nanoclusters, there may be many partially occupied states around the Fermi energy and thus adding one electron may not block the major plasmon decay channel or dramatically affect the dynamics.

**Figure 6:**
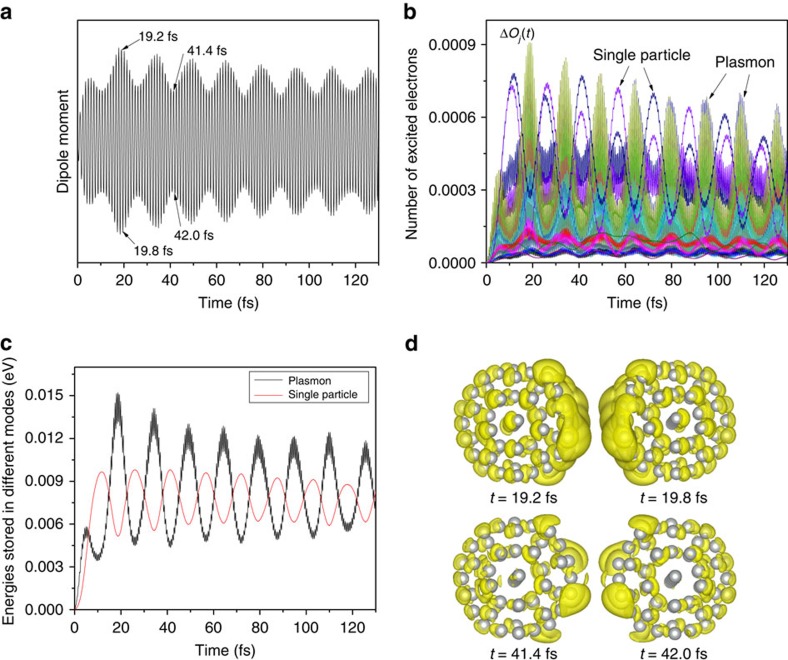
The time-dependent results for the nanocluster with a modified electronic structure. In this system, the single-particle *d* to *s* transitions resonant to the plasmon frequency do not exist. (**a**) The dipole moment exhibits a Rabi oscillation, and the amplitude of the dipole moment hardly decays. (**b**) The number of excited electrons Δ*O*_*j*_(*t*) also exhibits Rabi oscillations, and the major single-particle excitations are slightly off-resonant. (**c**) The energies stored in the plasmon mode and single-particle excitations exhibit similar Rabi oscillations. The energy is transferred back and forth between the plasmon and single-particle excitations, and the average energies stored in the plasmon and single-particle excitations do not change significantly with time. (**d**) The charge density difference Δ*ρ* at time *t* as labelled in **a**, which corresponds to the local maximum or local minimum of the dipole moment. When more energy is stored in the plasmon mode (19.2 and 19.8 fs), there are significant charge sloshings on the surface; however, when more energy is stored in the single-particle excitations (41.4 and 42.0 fs), there are less charge sloshings on the surface.

In 
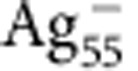
, the resonant single-particle *d* to *s* excitations have not been completely blocked (the *d* to 304th excitation exists). To change the system more dramatically, one may change the plasmon or single-particle frequencies. Experimentally, it can be realized by alloying with other metals, changing the nanocluster size/shape, or using some dielectric mediums. Theoretically, by further modifying the pseudopotential (see Methods section), we can artificially push down the *d*-state energies, making the *d* to *s* transition energies much greater than the plasmon frequency. Thus, all the single-particle *d* to *s* excitations become impossible. First, we observe an intriguing oscillation of the dipole amplitude (Fig. 6a). Δ*O*_*j*_(*t*) (Fig. 6b) still shows some single-particle excitations, from the 293rd to 332nd and 333rd eigen states (*s* to *p* excitations). However, their transition energies are slightly off-resonant from the plasmon frequency by ∼0.2 eV, and Δ*O*_*j*_ exhibits Rabi oscillations at the frequency Δ*ω*_*j*,*i*_−*ω*_p_. We also observe a cyclic energy transfer between the plasmon and single-particle excitations at the Rabi frequency (Fig. 6c). Different from the 
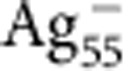
 case, the average energies stored in the plasmon and single-particle excitations do not change significantly with time. The charge density differences Δ*ρ* (Fig. 6d) at *t*=19.2 and 19.8 fs (more energy is stored in the plasmon) show stronger charge localization on the surface than those at *t*=41.4 and 42.0 fs (more energy is stored in the hot carriers). Around 40 fs, there is still charge localized on surface, because the plasmon is not fully decayed. Although Rabi oscillations in two-level systems are well known, it is interesting to observe Rabi oscillations between a collective plasmon mode and individual hot carriers. In the original Ag_55_ (Fig. 4b and Supplementary Fig. 4), we also observe some weak oscillations. However, the regular and complete energy back-flow from the single-particle excitations to the plasmon, as described by the resonant Rabi oscillation, is not observed. In plasmonic systems, when the plasmon resonance overlaps with the single-particle interband transitions, the plasmon becomes highly damped and *vice versa*. This conclusion was achieved by previous model system calculations^46^, and agrees with our results. The predicted Rabi oscillation may be observed experimentally using the real-time optical technique as described in the recent work by Mittal *et al*.^36^, where long-living (up to 80 fs) polarization beatings due to two plasmon modes were measured.

### Phonon effects

**Figure 7:**
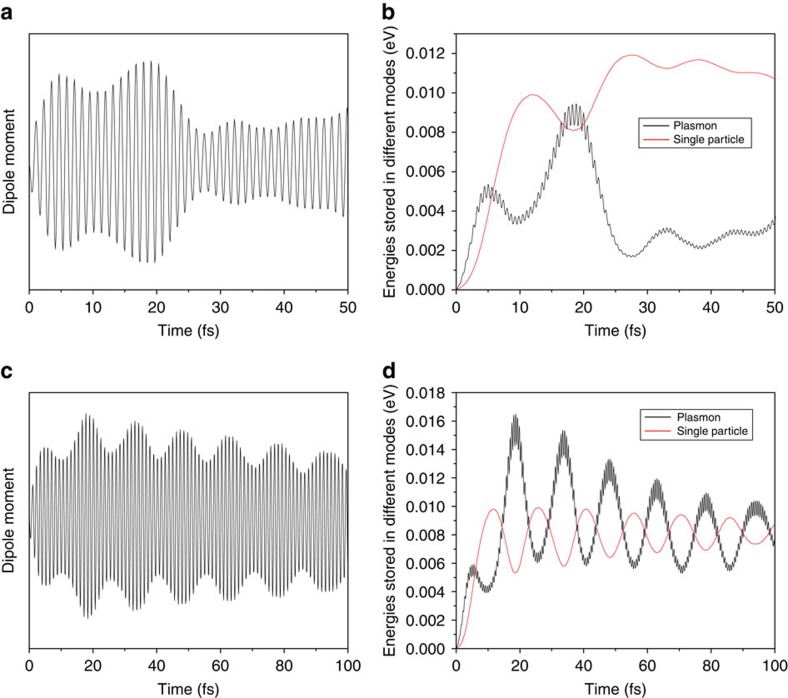
The rt-TDDFT Ehrenfest dynamics simulations at 50 K for neutral Ag_55_ (**a**,**b**) and Ag_55_ with the modified electronic structure **(c**,**d)**. In these simulations, the electron–phonon interactions are taken into account explicitly. (**a**) The dipole moment of the neutral Ag_55_ is similar to the result without electron–phonon effects shown in Fig. 2a. (**b**) The energies stored in the plasmon mode and single-particle excitations in the neutral Ag_55_ are also similar to the results without electron–phonon effects. It should be compared with Fig. 4b. (**c**) The dipole moment of the Ag_55_ with the modified electronic structure (no resonant single-particle *d* to *s* excitations). The dipole moment exhibits the Rabi oscillation, similar to the case without electron–phonon effects shown in Fig. 6a. (**d**) The energies stored in the plasmon mode and single-particle excitations also exhibit the Rabi oscillations when no resonant single-particle excitations exist. It should be compared with Fig. 6c. All these results show that phonons play a minor role in the plasmon decay.

The simulations above have only included plasmon dampings through hot-carrier generations within TDDFT. We have not included the electromagnetic radiation. 47 shows that the radiative effect is very weak, especially for small Ag nanoclusters, and thus can be safely ignored. Another plasmon damping mechanism is the electron–phonon interactions. Previously people believed the electron–phonon interactions can only occur in the timescale of pico-second^11^. However, a recent theoretical work by Bernardi *et al*.^48^ showed that the phonon-assisted carrier scattering can occur in tens of femtoseconds for bulk systems. It is thus interesting to investigate the phonon-assisted damping in nanoclusters. We have performed the rt-TDDFT Ehrenfest dynamics simulations. These simulations include the atomic movements following the *ab initio* forces in the rt-TDDFT, and are able to describe the electron–phonon effects explicitly. The results for neutral Ag_55_ and Ag_55_ without resonant hot-carrier generation channels are shown in Fig. 7. Both the dipole moment and the energies stored in both modes are almost the same as those in the simulations without electron–phonon interactions, although the simulations with electron–phonon interactions indeed show slightly faster decays. Our results show that the electron–phonon interactions only have minor effects in the nanocluster. This is probably because in bulks the energy conservation can always be satisfied and low-energy phonons can induce electron transitions, but it is not the case in nanoclusters with discrete electronic states.

## Discussion

We have performed rt-TDDFT simulations on the Ag_55_ nanocluster. Our rt-TDDFT simulations include the electron–electron interactions and electron–phonon interactions (using the Ehrenfest dynamics). The plane-wave basis set allows accurate descriptions of excited states. We do not include the electromagnetic radiations because they are extremely small in Ag nanoclusters. The simulations are under DFT and Kohn-Sham orbital framework, and do not explicitly use many-body wave functions. We propose an approach to distinguish the plasmon from single-particle excitations based on band to band transition coefficients *C*_*j*,*i*_(*t*). We find that the collective plasmon consists of off-resonant transitions with *C*_*j*,*i*_(*t*) oscillating in their amplitudes at the plasmon frequency, whereas the single-particle excitation is characterized by a slowly varying *C*_*j*,*i*_(*t*). The disappearance of the *ω*=0 peak in the Fourier transform of *C*_*j*,*i*_(*t*) makes the plasmon-related transitions different from the conventional independent off-resonant transitions described by the single-particle time-dependent perturbation theory, and is a hallmark of the plasmon. In Ag_55_, the single-particle *d* to *s* excitations resonant to the plasmon frequency exist. The plasmon will decay and generate hot carriers, and significantly enhance the hot-carrier generation rates. The main hot-carrier generations are from the low-energy *d*-states to the *s*-states around the Fermi energy. When applying a high-intensity laser, we also observe some nonlinear high-energy excitations. When most hot-carrier generation channels are blocked in 
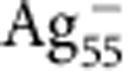
, the plasmon decays much slower. We also observe Rabi oscillations between the plasmon and single-particle excitations when the resonant single-particle *d* to *s* excitations have been completely blocked. Our rt-TDDFT Ehrenfest dynamic simulations show that the electron–phonon interactions do not play important roles in the plasmon decay in nanoclusters.

## Methods

### Computational setups

Our fast rt-TDDFT algorithm^31^ is employed within the local density approximation^49^. The norm-conserving pseudopotential is used and the valence electrons (Ag 4*d*^10^ and 5*s*) are expanded using plane-waves with an energy cutoff of 40 Ry. Although the plane-wave basis set significantly increases the computational cost compared with the local orbital basis set, it can describe the high-energy excited states and charge responses more accurately, which is critical for the plasmon study. The Brillouin zone integration is sampled with the Γ point only for the isolated Ag_55_ cluster. DFT cannot correctly predict the energies of the Ag *d*-states, which may affect the accuracy of the plasmon calculations^28^,^50^. To modify the energy of the Ag *d*-states, we add a Gaussian term to the *d*-channel of the Ag pseudopotential. We have fitted the pseudopotential so that the calculated density of states of the Ag *d*-bands agrees well with the experimental X-ray photoemission spectroscopy data^35^, as shown in Supplementary Fig. 1.

In experiments, the Ag_55_ adopts the icosahedral structure^32^,^33^. Therefore, we use the icosahedral model, and optimize the atomic coordinates within DFT to get the ground-state structure for the rt-TDDFT simulations. We have also calculated the truncated face-centred cubic structure of Ag_55_, and the simulated time-dependent results have similar features to those of the icosahedral structure, as shown in Supplementary Fig. 5 and Note 5. We have also calculated Au_55_, which is shown in Supplementary Fig. 6, but it does not have a clear plasmon peak.

In our simulations, we explicitly consider the atomic structure of the Ag_55_ nanocluster, and treat the Ag 4*d* electrons as valence electrons. Thus, our simulations give more accurate descriptions of the system than the studies based on jellium models.

When calculating the density of states, the DFT absorption spectrum, and *S*_C_(*ω*, *t*), we broaden the Dirac *δ* function using a Gaussian function with a s.d. of 0.1 eV.

### Plasmon decay channels

The rt-TDDFT simulation explicitly includes the electron–electron interaction, which is critical for the plasmon excitation and plasmon-induced hot-carrier generations. The rt-TDDFT does not include the radiative damping. Previous study shows that the radiative effect is very weak for small Ag nanoclusters, so it is safe to ignore it^47^. The electron–phonon interactions can be explicitly included by using the Ehrenfest dynamics (which is beyond the Born-Oppenheimer approximation). At first, we have fixed the atomic positions in the simulations to focus on the effects of electron–electron interactions. Previous studies show that the electron–phonon interactions are on the timescale of picoseconds^11^, and thus are not important for plasmons. In the last part (Fig. 7), we have included the atomic movements at 50 K using the Ehrenfest dynamics to show that the electron–phonon interactions only have minor effects. Such a low temperature is typical for Ag_55_ in experiments due to the high mobility of the nanocluster^33^.

### TDDFT algorithm and related formulas

In our rt-TDDFT simulations, the time-dependent electronic state *ψ*_*i*_(*t*) at *t*=0 is occupied according to the ground-state Fermi-Dirac occupation number 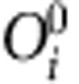, and the 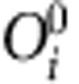 will not be changed with time. The time-dependent wave functions *ψ*_*i*_(*t*) are always orthogonal to each other. To evolve *ψ*_*i*_(*t*) with time, we expand it onto the adiabatic eigen states *φ*_*j*_(*t*) (which satisfies *H*[*ρ*(*t*), *t*]*φ*_*j*_(*t*)=*ω*_*j*_(*t*)*φ*_*j*_(*t*) for the time-dependent Hamiltonian *H*[*ρ*(*r*), *t*]):


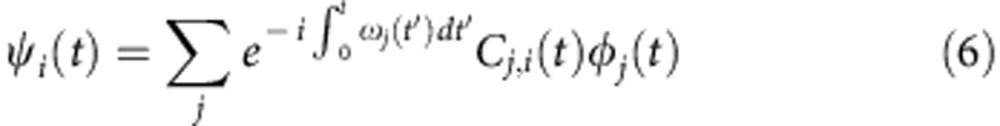


The *C*_*j*,*i*_ is the expansion coefficient which can be calculated using our rt-TDDFT algorithm^31^. The off-diagonal element *C*_*j*,*i*_ (*i*≠*j*) represents the transition from the *i*th adiabatic eigen state to the *j*th adiabatic eigen state. We note that *C*_*j*,*i*_ might depend on an arbitrary phase factor due to the non-unique definition from *φ*_*j*_(*t*) to *φ*_*j*_(*t*+*dt*). We have removed this arbitrary phase factor by requiring 
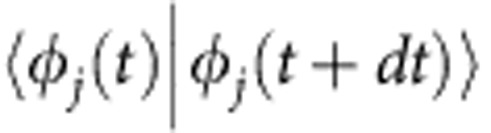
 to be real. Since *φ*_*j*_(*t*) can be taken as a real function (without magnetic fields), there will be no Berry phase. The simulation is under DFT and Kohn-Sham orbital framework, and thus it does not explicitly use many-body wave functions. By using our new algorithm, we can set the simulation time step to 0.1 fs (∼100 times greater than that in conventional rt-TDDFT simulations)^31^, which allows us to run long-time simulations using plane-wave basis sets. Note, in the Results part above, we have used constant eigen energies *ω*_*j*_ for the purpose of analysis, as the variations of *ω*_*j*_(*t*) are rather small.

The charge density is calculated by


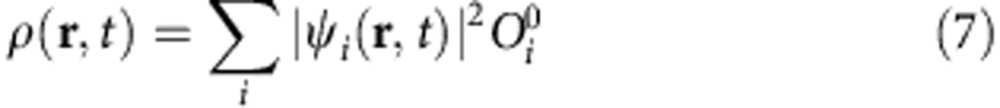


Using the coefficients *C*_*j*,*i*_, we define the occupation change Δ*O*_*j*_(*t*) on the *j*th adiabatic eigen state as:


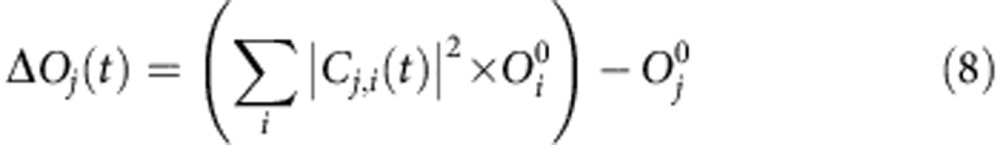


The Δ*O*_*j*_(*t*) represents the occupation change on the *j*th adiabatic eigen state at time *t* with respect to its ground-state occupation 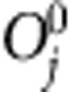 due to various electronic excitations. Δ*O*_*j*_(*t*) can be either positive or negative. Positive Δ*O*_*j*_(*t*) indicates increasing occupations (excited electrons) and negative Δ*O*_*j*_(*t*) indicates decreasing occupations (excited holes).

To simulate the laser excitations, we apply an external electric field to the Hamiltonian to mimic the laser pulse


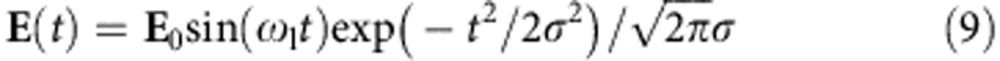


The electric field **E** is along the *x* axis. *σ*, *ω*_l_ and 
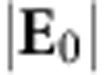
 define the duration, central frequency and amplitude of the electric field, respectively. In this study, we set *ω*_l_=*ω*_p_=3.6 eV, *σ*=6 fs and 
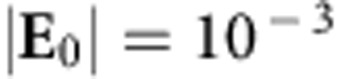
 Hartree per Bohr.

The polarizability can be obtained from the time-dependent dipole moment of the nanocluster^51^


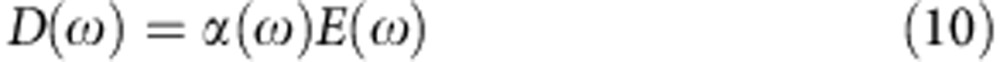


where *D*(*ω*) and *E*(*ω*) are the Fourier transforms of the dipole moment and the electric field, respectively. The absorption spectrum is obtained from the imaginary part of *α*(*ω*).

The energies stored in the plasmon and the single-particle (hot-carrier) modes are estimated as


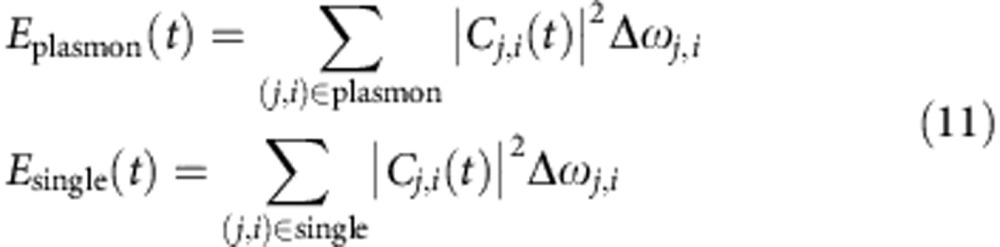


where ‘(*j*, *i*)∈plasmon' and ‘(*j*, *i*)∈single' indicate the *C*_*j*,*i*_(*t*) belongs to the rapidly oscillating transitions and the slowly varying transitions, respectively. For a given (*j*, *i*) pair, the *C*_*j*,*i*_(*t*) either belongs to the plasmon or single particle, and there is no mixture between the two types. Thus the energies in equation (11) can be calculated unambiguously.

## Additional information

**How to cite this article:** Ma, J. *et al*. Interplay between plasmon and single-particle excitations in a metal nanocluster. *Nat. Commun.* 6:10107 doi: 10.1038/ncomms10107 (2015).

## Supplementary Material

Supplementary Information Supplementary Figures 1-6, Supplementary Notes 1-5 and Supplementary References
