# Supplementary material for: Interplay between plasmon and single-particle excitations in a metal nanocluster
Source: Nat Commun. 2015 Dec 17;6:10107. doi: 10.1038/ncomms10107 (PMC4703846; doi:10.1038/ncomms10107)
Supplement: Supplementary Information — Supplementary Figures 1-6, Supplementary Notes 1-5 and Supplementary References [file ncomms10107-s1.pdf]

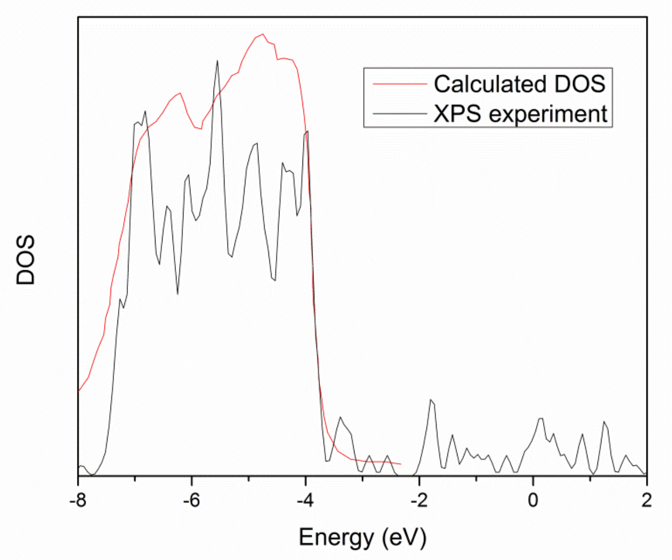

Supplementary Figure 1. The comparison of the calculated density of states (DOS) of the bulk Ag with the experimental result. The red line is the experimental X-ray photoemission (XPS) data for the bulk Ag extracted from Fig. 2 of Ref. 1. We have modified the pseudopotential so that the calculated energy of the Ag *d*-band (black line) is in good agreement with the experimental data.

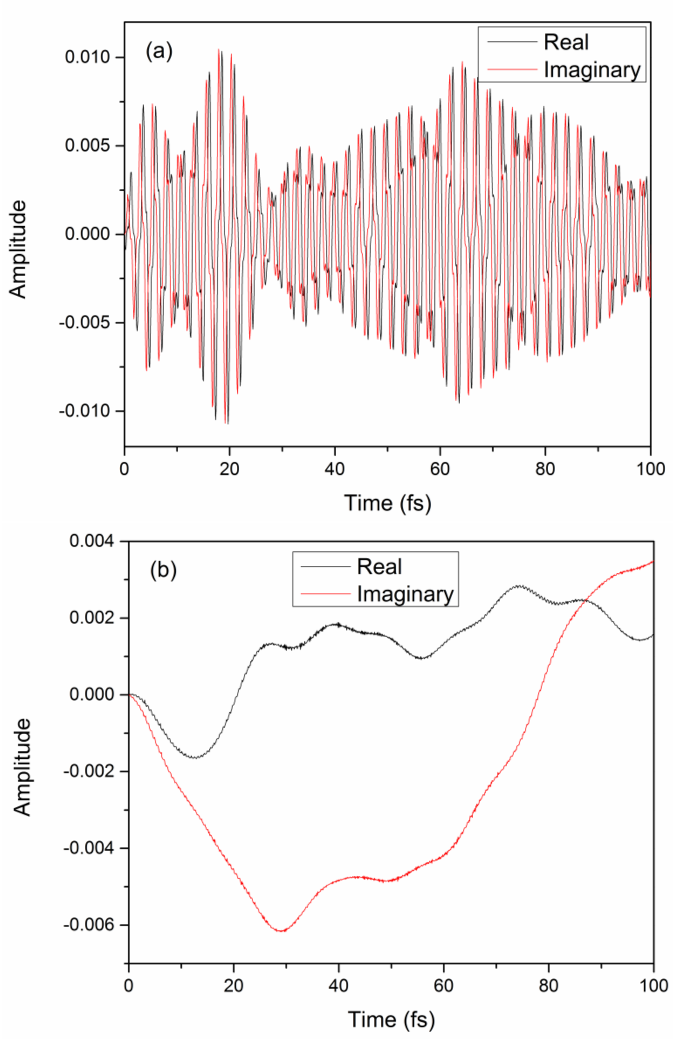

Supplementary Figure 2. The real and imaginary parts of $C_{j,i}(t)$. (a) The $C_{j,i}(t)$ for a typical rapidly-oscillating transition. Both the real and imaginary parts of $C_{j,i}(t)$ oscillate around zero. The averages of both the real and imaginary parts are zero, and thus the Fourier transform of $C_{j,i}(t)$ has no peak at $\omega = 0$. (b) The $C_{j,i}(t)$ for a typical slowly-varying transition. Different from (a), both the real and imaginary parts of $C_{j,i}(t)$ vary smoothly.

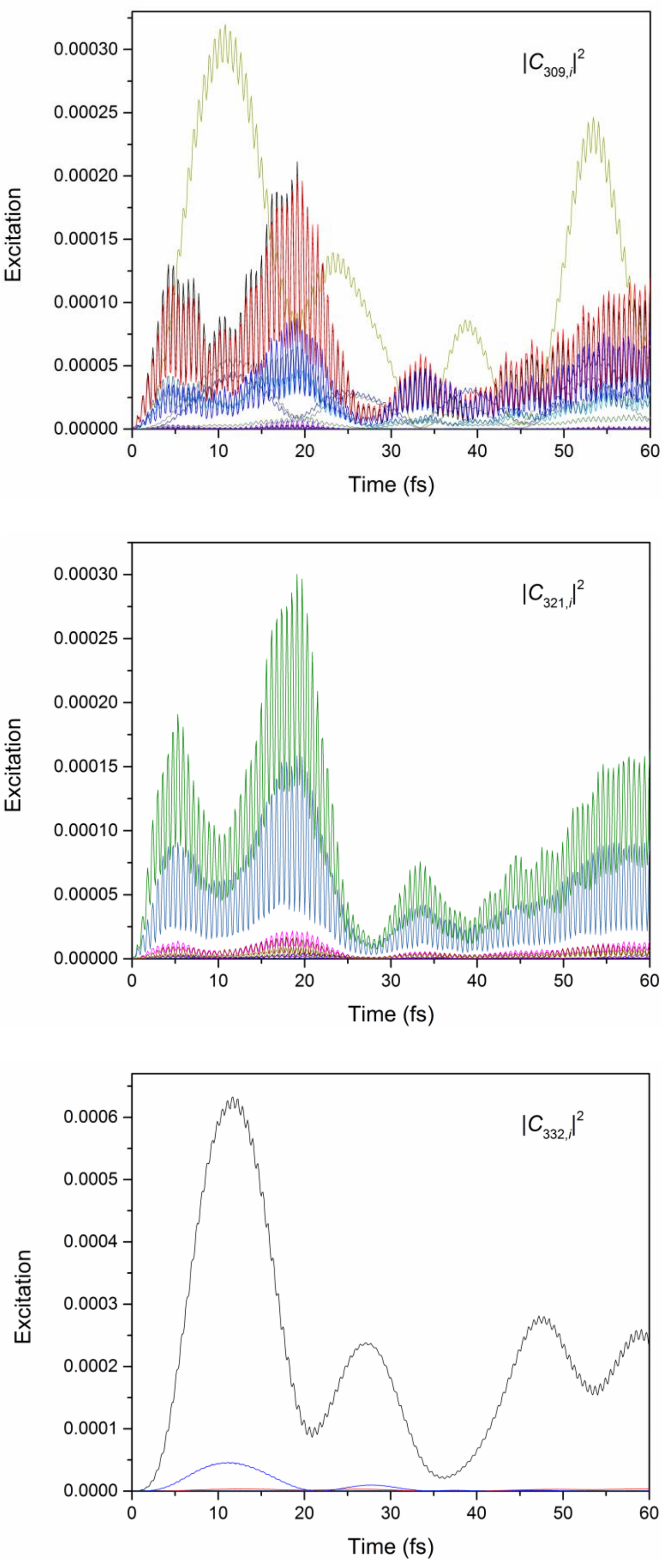

Supplementary Figure 3. The $\left|C_{j,i}(t)\right|^2$ for $j = 309$, 321, and 332 eigen states. All these $\left|C_{j,i}(t)\right|^2$ can be classified in the two distinct types as discussed in the manuscript.

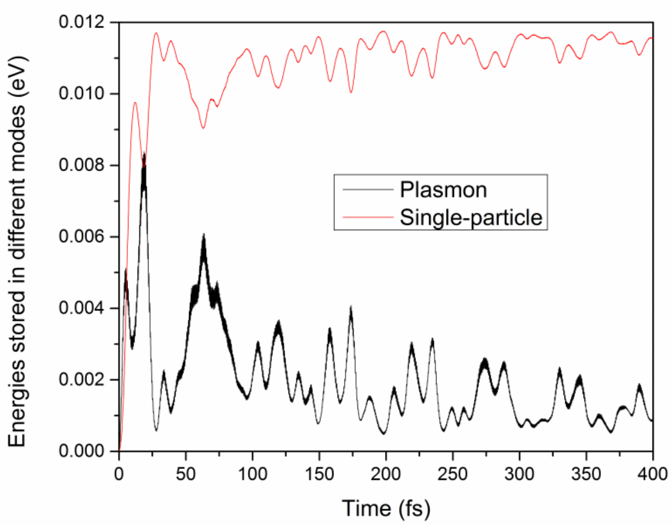

Supplementary Figure 4. The energies stored in the plasmon and single-particle excitations in Ag_{55}. The simulation has been performed for 400 fs. Almost all the energies stored in the plasmon are transferred to the single-particle (hot-carrier) excitations, although there are small energy oscillations between the plasmon and hot-carriers.

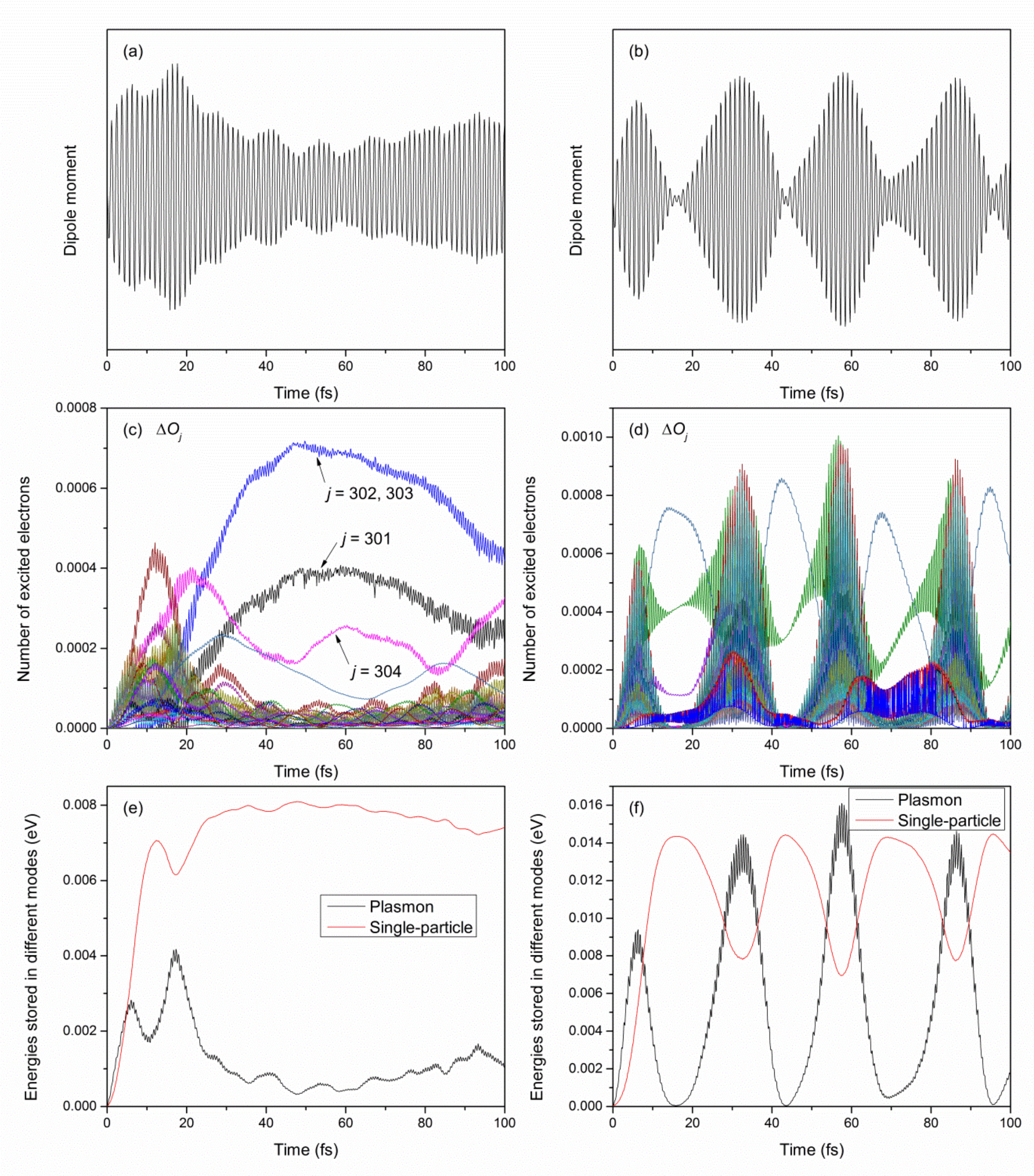

Supplementary Figure 5. The time-dependent simulations for the truncated fcc structure of Ag_{55} nanocluster. The left panels show the results when the single-particle *d*-to-*s* excitations resonant to the plasmon frequency exist, and the right panels show the results when those resonances do not exist. (a) When the resonant excitations exist, the dipole moment decays with time; (c) the major plasmon decay is through the hot-carrier generations from the low-energy *d*-states to the states around the Fermi energy (several important states are labeled in the panel); and (e) the energies stored in the plasmon are mostly transferred to hot-carriers. (b) When the resonant excitations do not exist, the dipole moment shows Rabi oscillations; (d) the numbers of excited electrons on eigen states exhibit the same Rabi oscillations; and (f) the energies oscillate between the plasmon mode and hot-carriers at the Rabi frequency. All these results are similar to those of the icosahedral Ag_{55} nanocluster described in the manuscript.

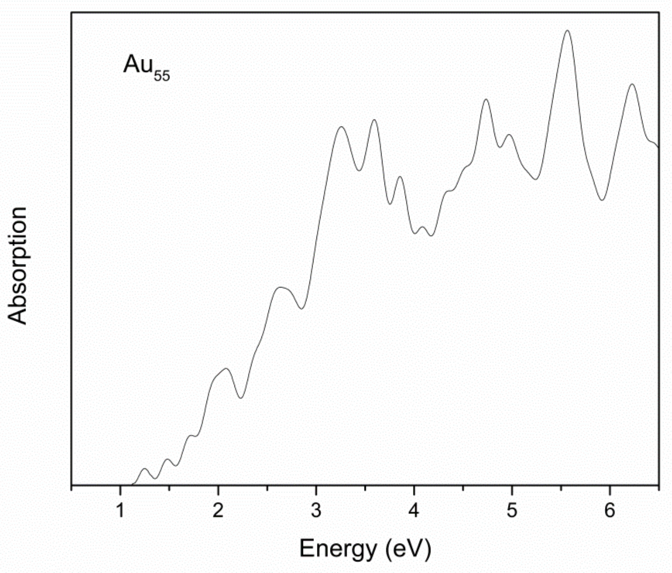

Supplementary Figure 6. The calculated absorption spectrum of the Au_{55} nanocluster. The absorption spectrum does not show a clear plasmon peak as in the Ag_{55} case.

Supplementary Note 1. Correcting the Ag *d*-band energy

The local density approximation functional overestimates the Ag *d*-band energy, which can cause error in the simulation. To correct the error, we have modified the Ag pseudopotential by adding a Gaussian term to the *d*-channel, so that the calculated *d*-band energy of the bulk Ag is in good agreement with the experiment. The calculated DOS and the experimental XPS spectrum of the bulk Ag are shown in Supplementary Fig. 1.

Supplementary Note 2. The real and imaginary parts of the transition coefficients

Supplementary Figure 2 shows the real and imaginary parts of $C_{j,i}(t)$ for a typical rapidly-oscillating transition (a) and those for a slowly-varying transition (b). All other transitions have similar features. For rapidly-oscillating transitions, both the real and imaginary parts of $C_{j,i}(t)$ oscillate rapidly around 0. The averages of the $C_{j,i}(t)$ are always 0, and thus the Fourier transforms of $C_{j,i}(t)$ have no peak at $\omega = 0$. In contrast, the real and imaginary parts of $C_{j,i}(t)$ of the slowly-varying transitions change smoothly and the averages are not zero, so the Fourier transforms should have a peak at $\omega = 0$. The results show that the zero-frequency peak in the Fourier transforms of $C_{j,i}(t)$ is a clear signal for distinguishing the two different types of transitions.

Supplementary Note 3. The transition coefficients for other states

Supplementary Figure 3 shows the $\left|C_{j,i}(t)\right|^2$ for other eigen states ($j = 309$, 321, and 332). All these $\left|C_{j,i}(t)\right|^2$ can be classified in the two distinct types discussed in the manuscript. For most eigen states, there exist both the rapidly-oscillating $C_{j,i}$ (plasmon) and the slowly-varying $C_{j,i}$ (single-particle), which are similar to the cases of the eigen states $j = 304$ and 309. In other words, the excited electrons (holes) on most eigen states are generated by both the plasmon and single-particle excitations. For several eigen states, there only exist the rapidly-oscillating $C_{j,i}$, such as $j = 321$, or the slowly-varying $C_{j,i}$, such as $j = 332$. For these eigen states, the plasmon or the single-particle excitations dominate.

Supplementary Note 4. Long-time TDDFT simulation

Supplementary Figure 4 shows the energies stored in the plasmon and single-particle excitations in the Ag_{55} for a long-time (400 fs) simulation. Almost all the energy stored in the plasmon is transferred to the single-particle (hot-carrier) excitations and no significant energy back-flow is observed, although there are small energy transfers. It shows the plasmon can enhance the final hot-carrier generations, and most absorbed solar energy can be used for photovoltaic or photochemical applications by collecting the plasmon-generated hot-carriers.

Supplementary Note 5. Other nanoclusters

Besides the icosahedral structure (the most stable structure of Ag_{55} in experiments), we have also calculated the excitations and energy transfers in a truncated face-centred cubic (fcc) structure of Ag_{55}, and the results are shown in Supplementary Fig. 5. When the single-particle *d*-to-*s* excitations resonant to the plasmon frequency exist (panels a, c, and e), the plasmon decays through the hot-carrier generations from the low-energy *d*-states to the states around the Fermi energy (which are labeled in panel c), and almost all the energies stored in the plasmon are transferred to hot-carriers; when those resonant excitations do not exist (panels b, d, and f), the dipole moment, the numbers of excited electrons, and the energies stored in the plasmon mode and hot-carriers all exhibit Rabi oscillations. All these results are similar to those of the icosahedral Ag_{55} nanocluster described in the manuscript.

We have also calculated the Au_{55} nanocluster. The calculated absorption spectrum is shown in Supplementary Fig. 6. Different from the Ag nanocluster, Au_{55} does not have a clear plasmon peak, and thus we cannot study the plasmon generation and decay clearly. Our absorption spectrum of the Au cluster is in agreement with previous calculations (e.g., Fig. 2 of Ref. 2).

Supplementary References

1. Barrie, A. & Christensen, N. E. High-resolution X-ray photoemission spectra of silver. *Phys. Rev. B* **14**, 2442-2447 (1976).
2. Iida, K., Noda, M., Ishimura, K. & Nobusada, K. First-principles computational visualization of localized surface plasmon resonance in gold nanoclusters. *J. Phys. Chem. A* **118**, 11317-11322 (2014).
